# Preclinical stem cell therapy in fetuses with myelomeningocele: A systematic review and meta‐analysis

**DOI:** 10.1002/pd.5887

**Published:** 2021-01-11

**Authors:** Yada Kunpalin, Sindhu Subramaniam, Silvia Perin, Mattia F. M. Gerli, Jan Bosteels, Sebastien Ourselin, Jan Deprest, Paolo De Coppi, Anna L. David

**Affiliations:** ^1^ Elizabeth Garrett Anderson Institute for Women's Health University College London London UK; ^2^ Department of Development and Regeneration, Cluster Woman and Child Biomedical Sciences KU Leuven Leuven Belgium; ^3^ Great Ormond Street Institute of Child Health University College London London UK; ^4^ Division of Surgery and Interventional Science Royal Free Hospital University College London London UK; ^5^ Cochrane Belgium Belgian Centre for Evidence‐Based Medicine (Cebam) Leuven Belgium; ^6^ School of Biomedical Engineering & Imaging Sciences King's College London London UK; ^7^ Department of Obstetrics and Gynaecology University Hospitals Leuven Leuven Belgium

## Abstract

**Objective:**

We performed a systematic review to summarize the efficacy and safety of in utero stem cells application in preclinical models with myelomeningocele (MMC).

**Methods:**

The study was registered with PROSPERO (CRD42019160399). We searched MEDLINE, Embase, Web of Science, Scopus and CENTRAL for publications articles on stem cell therapy in animal fetuses with MMC until May 2020. Publication quality was assessed by the SYRCLE's tool. Meta‐analyses were pooled if studies were done in the same animal model providing similar type of stem cell used and outcome measurements. Narrative synthesis was performed for studies that could not be pooled.

**Results:**

Nineteen and seven studies were included in narrative and quantitative syntheses, respectively. Most used mesenchymal stem cells (MSCs) and primarily involved ovine and rodent models. Both intra‐amniotic injection of allogeneic amniotic fluid (AF)‐MSCs in rat MMC model and the application of human placental (P)‐MSCs to the spinal cord during fetal surgery in MMC ovine model did not compromise fetal survival rates at term (rat model, relative risk [RR] 1.03, 95% CI 0.92–1.16; ovine model, RR 0.94, 95% CI 0.78–1.13). A single intra‐amniotic injection of allogeneic AF‐MSCs into rat MMC model was associated with a higher rate of complete defect coverage compared to saline injection (RR 16.35, 95% CI 3.27–81.79). The incorporation of human P‐MSCs as a therapeutic adjunct to fetal surgery in the ovine MMC model significantly improved sheep locomotor rating scale after birth (mean difference 5.18, 95% CI 3.36–6.99).

**Conclusions:**

Stem cell application during prenatal period in preclinical animal models is safe and effective.

## Key Points

What's already known about this topic?

- Myelomeningocele (MMC) is a severe congenital malformation of the central nervous system causing lifelong sensory and motor impairments, bowel and bladder dysfunctions, and orthopaedic disabilities
- Fetal surgery for MMC reduces ventriculoperitoneal shunt requirement, increase the ability to walk of the affected children.

What does this study add?

- Safety and efficacy evidence of in utero stem cell application in preclinical MMC settings
- The application of in utero mesenchymal stem cells is safe and effective in inducing defect coverage and improve motor function in small and large animal models, respectively

## 1 INTRODUCTION

Myelomeningocele (MMC) is a severe congenital malformation of the central nervous system resulting from an incomplete closure of the neural tube during the third–fourth week of embryonic development.^1^ The prevalence of MMC varies greatly among geographical areas ranging from 0.3 to 59.0 cases per 10,000 births.^2^ MMC is characterised by the protrusion of the neural placode and its meninges through a malformed vertebral arch and skin defect. The condition can be detected by prenatal ultrasound scan as early as the first trimester; however, the majority of cases are diagnosed during the second trimester (anomaly) ultrasound scan.^3^, ^4^ Apart from preventive therapy using periconceptual vitamins such as folic acid, current management following prenatal diagnosis may include termination of pregnancy, postnatal or more recently fetal surgery.^5^ The rationale for fetal repair before birth is that MMC is a ‘progressive’ condition with cumulative spinal cord functional loss throughout gestation, as demonstrated in clinical and animal studies.^6^, ^7^, ^8^ Fetal surgery can arrest this deterioration and improve the patients' ability to walk unaided at 30‐month old.^9^, ^10^, ^11^ However, the benefit of the surgery to bladder function is still under review.^12^, ^13^, ^14^, ^15^, ^16^ Despite these improvements, there are several shortcomings of fetal surgery. Although the number of centres offering fetal surgery for MMC has been increasing,^17^ global availability is still limited. Furthermore, fetal surgery is usually performed in the late second trimester, between 23 and 26 weeks' gestation, to reduce the risk of chorioamniotic membrane separation and associated preterm birth.^18^, ^19^ Moreover, fetal surgery is not a cure. When considering patient outcomes at 30‐month‐old age; for example, approximately half of the fetal treated patients have to rely on clean intermittent catheterization to pass urine and more than half cannot walk without the aid of orthosis.^11^, ^12^

Additional interventions during fetal life such as the use of stem cells, may improve the shortcomings of fetal surgery. Stem cell transplantation, particularly of mesenchymal stem cells (MSCs), have been reported in both animal and clinical studies for spinal cord injury.^20^, ^21^, ^22^ In clinical cases of individuals suffering from spinal cord injury, stem cell therapy improves light‐touch and pinprick sensory function, bladder function and also increases the score of the daily living activities when compared to patients who receive only rehabilitation.^22^ For treatment of MMC, in utero stem cell therapy has been reported to improve outcome in several animal studies, but as yet no human trials have been conducted.

Several animal models have been used to evaluate pathophysiology and treatment options for MMC. These models can be divided into surgically and non‐surgically induced models. All ovine, rabbit and chick models involve surgical manipulation; laminectomy and resection of dura mater, to create an MMC‐like lesion.^23^, ^24^ In contrast, in the rat model, the lesion is induced by gavaging retinoic acid to pregnant dams early in gestation. Retinoic acid is a well‐known teratogen that disrupts the process of neural tube closure leading to the MMC defect in the pups.^25^ All of the aforementioned models, both surgical and non‐surgical, have been applied to study feasibility, safety and efficacy of in utero stem cell transplantation for MMC.

In this study, we systematically reviewed the application of stem cells in preclinical animal models of MMC with regards to their safety, efficacy and to justify the possibility of translation into a clinical study.

## 2 MATERIALS AND METHODS

This systematic review was conducted according to the Preferred Reporting Items for Systematic Review and Meta‐analyses guidelines (www.prisma-statement.org).^26^ Our protocol was registered with the International Prospective Register of Systematic Reviews (PROSPERO; CRD42019160399) before commencement.

### 2.1 Literature search strategy

An electronic literature search was performed in MEDLINE (PubMed), Embase, Web of Science, Scopus and the Cochrane Library from inception until May 2020. The search strategy included both Medical Subject Headings term and free text words (Data S1). Topic‐related reviews were manually searched to retrieve additional relevant articles. Endnote X9 (Thomson Reuters) was used to remove duplicate studies based on names of the authors, titles, and year of publications.

### 2.2 Inclusion and exclusion criteria

The population was MMC animals receiving an in vivo*,* in utero application of stem cells. The intervention included any type of stem cells; embryonic stem cells (ESCs), pluripotent stem cells (IPSCs), neuronal stem cells (NSCs), neural crest stem cells (NCSCs) and MSCs. Comparator group was animals receiving only fetal surgery, saline injection or no treatment at all. Studies were excluded if stem cells were administered after birth or was published in non‐English language. Outcomes examined were related to safety, survival and efficacy as described below. No date restrictions were applied. Editorial comments, review studies and publications without full‐text accessibility were excluded.

### 2.3 Study selection

Titles and abstracts were independently screened and selected for relevance by two reviewers (Yada Kunpalin and Sindhu Subramaniam). A full‐text review was performed for all the selected studies based on the aforementioned criteria. Any disagreement was resolved through discussion with a third reviewer (Silvia Perin). In case of overlapping studies, only the most recent publication was included.

### 2.4 Data extraction

A predefined pro forma was created by the reviewers before data extraction. Extracted information included year of publication, types of animal model, number of animals, sample randomization and gestation age (GA) when the defect was created. Treatment information included source and types of stem cells, dosage, type of vehicles, controls and GA when stem cells were administered, and GA at euthanasia. Extracted outcomes were animal survival rate, defect coverage, spinal cord histopathology and neurological function. Corresponding authors were contacted for further/missing data.

### 2.5 Quality appraisal

Risk of bias was independently assessed by Yada Kunpalin and Sindhu Subramaniam by the Systematic Review Centre for Laboratory Animal Experimentation's (SYRCLE's) tool for animal interventional studies.^27^ Discrepancies between the reviewers were resolved through consensus by the third reviewer (Silvia Perin).

### 2.6 Data synthesis and statistical methods

Meta‐analyses were performed only if studies were consistent with regards to the type of animal model, stem cells and outcome measurements. For studies that could not be pooled, we present a narrative data synthesis with descriptive statistics.

Meta‐analyses were carried out using the software provided by the Cochrane Collaboration, Review Manager (RevMan) version 5.3. Quantification of the heterogeneity across the included studies was assessed by chi‐squared value test and inconsistency index (I^2^). I^2^ of >50% and <0.1 of *α* value of chi‐square were deemed to have significant heterogeneity.^28^ Consequently, a random‐effect model was used to analyse the data; otherwise, the fixed‐effect model was applied. In terms of animal survival rate and MMC defect coverage rate, the results were represented by relative risk (RR). For Sheep Locomotor Rating (SLR) scale, the improvement was displayed with mean difference.

## 3 RESULTS

### 3.1 Study selection

**FIGURE 1:**
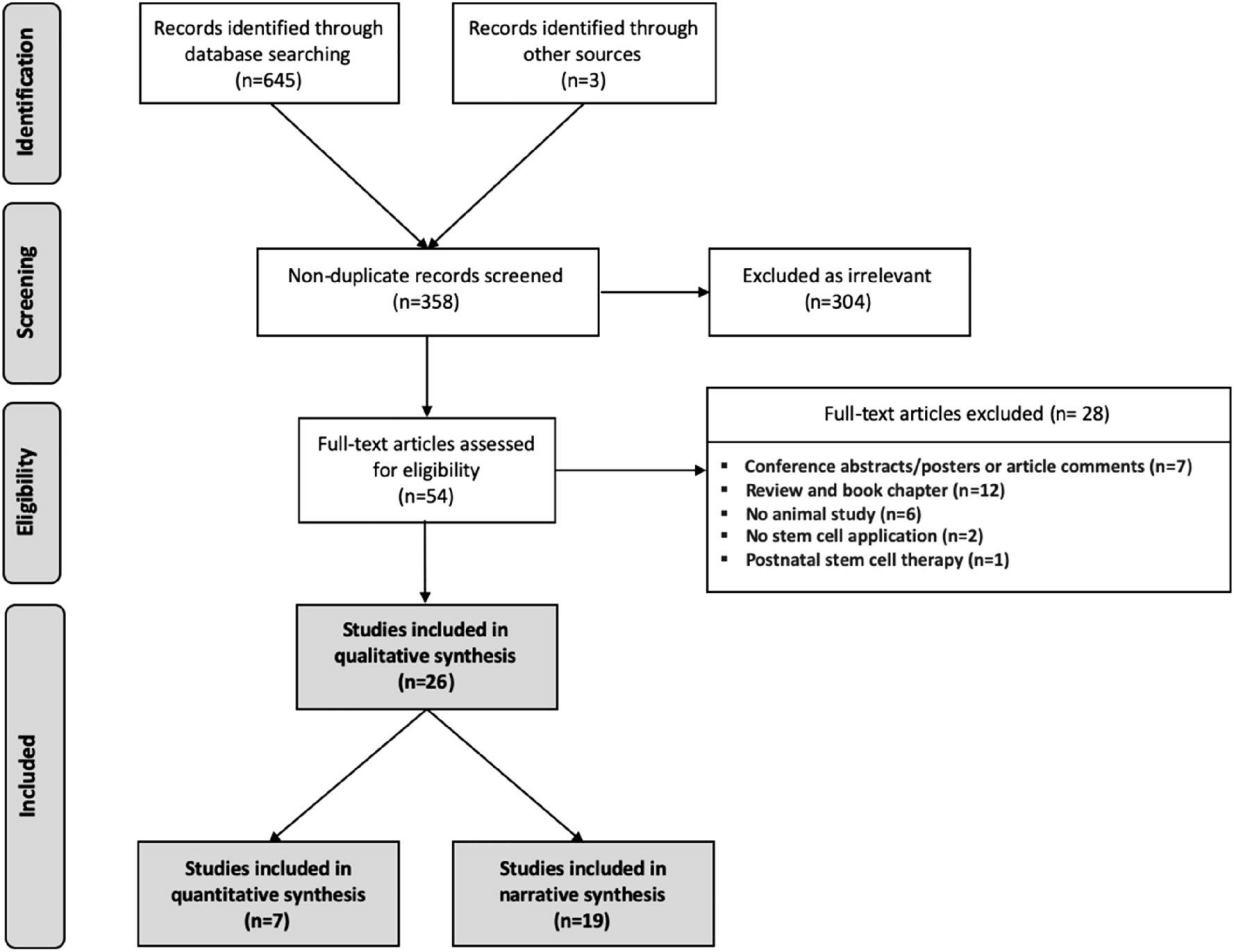
Flow diagram of illustrated study selection (adapted from preferred reporting items for systematic reviews and meta‐analysis [PRISMA])^24^

Electronic and manual search yielded 648 records published from inception until May 2020; 86 from MEDLINE (PubMed), 217 from Embase, 132 from Web of Science, 210 from Scopus and none from the Cochrane Library. Additional records were retrieved from manual search of reference lists and directly from previous publications of research groups. After removing duplicates, the remaining 358 records were screened for relevant titles and abstracts. Of these, 304 records were excluded as irrelevant (Figure 1). A total number of 54 records were reviewed as full‐text, of which 26 studies were included in the qualitative synthesis. Reasons for exclusion were insufficient information (conference abstract/poster presentations or article comments (25%, 7/28), inadequate study design (review/book chapter) (43%, 12/28), no in vivo animal study included (21%, 6/28), no stem cells application (7%, 2/28) and postnatal stem cell therapy only (4%, 1/28; Figure 1).

### 3.2 Risk of bias assessment

**FIGURE 2:**
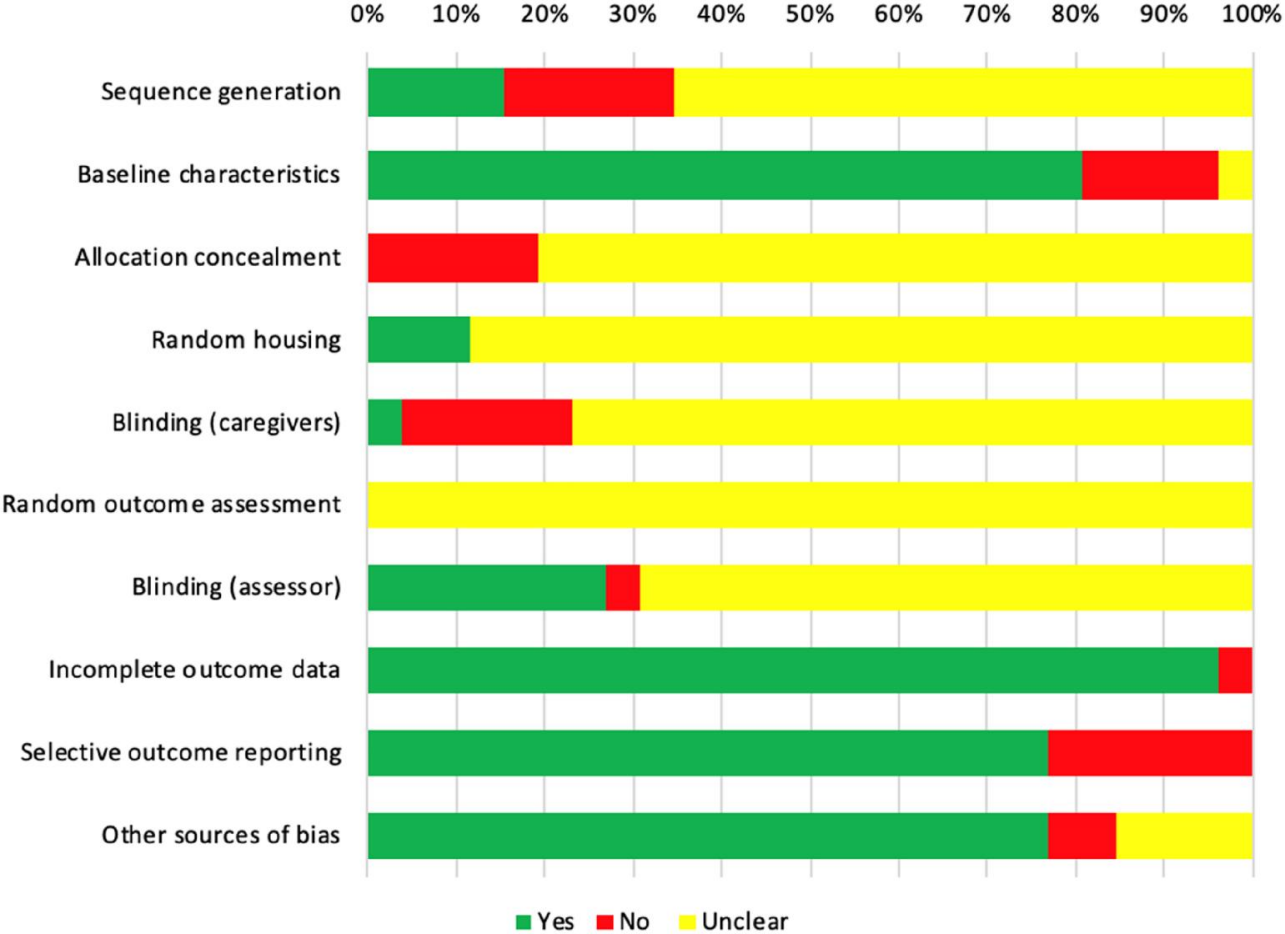
Risk of bias assessment by SYRCLE's risk of bias tool for animal studies^25^

Risk of bias of the included studies is shown in Figure 2. The majority of the studies had a high risk of bias owing to selective outcome reporting (23.1%, 6/26), inadequate description of sequence generation (19.2%, 5/26), allocation concealment (19.2%, 5/26) and caregiver/researcher blinding (19.2%, 5/26). None of the included studies completely described information regarding animal housing and/or random/blinding method for outcome assessment as per recommended by ARRIVE guidelines.^29^

### 3.3 Study characteristics

The characteristics of the included studies, such as type and source of stem cells, animal models and available outcomes, are shown in Table 1.^30^, ^31^, ^32^, ^33^, ^34^, ^35^, ^36^, ^37^, ^38^, ^39^, ^40^, ^41^, ^42^, ^43^, ^44^, ^45^, ^46^, ^47^, ^48^, ^49^, ^50^, ^51^, ^52^, ^53^, ^54^, ^55^ Most studies used MSCs (77%, 20/26), with the placenta, amniotic fluid and bone marrow as the source of cells. Xenogeneic stem cell transplantation with human cells (ESCs, NCSCs, bone marrow [BM]‐MSCs, amniotic fluid [AF]‐MSCs, placental [P]‐MSCs) was performed in almost half of the studies (46%, 12/26). The majority of animal models studied were rat strains (58%, 15/26; Wistar, Sprague Dawley or Lewis) all of which had MMC created using retinoic acid (40 or 60 mg/kg). Studies in ovine (27%, 7/26) all used surgical creation of MMC between 75 and 112 days GA. Chicken embryo was assessed in three studies (11%, 3/26) with MMC created surgically at Hamburger and Hamilton stage 18–19. One study was performed in the rabbit (4%, 1/26) with MMC surgically created at E18‐19 days. All included studies evaluated animals immediately after term delivery and/or up to 24 h after birth.

**TABLE 1:** Characteristics of included studies

Study	Stem cells			Animal models			Stem cell application			Results of stem cell application			
	Donor	Type	Source	Animal	Lesion induction	Timing of lesion induction	Dosage	Timing of transplantation	Vehicle	Timing of evaluation	Animal survival	Defect coverage	Spinal cord changes
Lee, 2004^(30)^	Human	ESCs	Blastocyst	Chicken embryo	Surgical creation	HH18–19	2 × 10^4^ cells/amniotic cavity	HH18‐19; immediately after surgical creation	Intra‐amniotic injection	HH 28, 30 and 35	Yes	Yes	No
Lee, 2006^(31)^	Human	ESCs	Blastocyst	Chicken embryo	Surgical creation	HH18–19	4 × 10^4^ cells/amniotic cavity	HH18–19; immediately after surgical creation	Intra‐amniotic injection	HH 30, 35 and 40	Yes	Yes	No
Lee, 2010^(32)^	Human	NSCs and MSCs	Fetal NCSCs, telencephalon (GA 14‐18w); fetal MSCs, spinal BM (GA 12‐ 15w)	Chicken embryo	Surgical creation	HH18–19	2 × 10^4^ cells/amniotic cavity	HH18–19; immediately after surgical creation	Intra‐amniotic injection	HH 28, 30 and 35	Yes	Yes	No
Li, 2012^(33)^	Adult Wistar rat	MSCs	Bone marrow	Wistar rat	Retinoic acid (140 mg/kg)	E10	1.4–7 x10^3^ cells/spinal cord	E16‐18	Direct spinal cord injection	E20	Yes	No	No
Li, 2014^(34)^	Adult Wistar rat	MSCs	Bone marrow	Wistar rat	Retinoic acid (140 mg/kg)	E10	6–10 × 10^3^ cells/spinal cord	E16	Direct spinal cord injection	E20	No	No	Yes
Ma, 2015^(35)^	Adult Wistar rat	MSCs	Bone marrow	Wistar rat	Retinoic acid (140 mg/kg)	E10	6–10 × 10^3^ cells/spinal cord	E16	Direct spinal cord injection	E20	Yes	No	Yes
Li, 2016^(36)^	Adult Wistar rat	MSCs	Bone marrow	Wistar rat	Retinoic acid (140 mg/kg)	E10	2 × 10^7^ cells/spinal cord	E16	Chitosan‐gelatin scaffold	E20	Yes	Yes	No
Wei, 2020a^(37)^	Adult Wistar rat	MSCs	Bone marrow	Wistar rat	Retinoic acid (140 mg/kg)	E10	4 – 6 x 10^6^ cells/uterine cavity	E16	Intra‐amniotic injection and	E21	Yes	Yes	No
Wei, 2020b^(38)^	Adult Wistar rat	MSCs	Bone marrow	Wistar rat	Retinoic acid (140 mg/kg)	E10	5 × 10^6^ cells/uterine cavity	E15	Ex vivo intra‐amniotic injection	E20	Yes	Yes	Yes
Dionigi, 2015a^(39)^	Normal Lewis rat fetus	MSCs	Amniotic fluid (E21)	Sprague‐Dawley rat	Retinoic acid (60 mg/kg)	E10	1 × 10^5^ cells/uterine cavity	E17	Intra‐amniotic injection	E21	Yes	Yes	No
Dionigi, 2015b^(40)^	Normal Lewis rat fetus	MSCs	Amniotic fluid (E21)	Sprague‐Dawley rat	Retinoic acid (60 mg/kg)	E10	1 × 10^5^ cells/uterine cavity	E17	Intra‐amniotic injection	E21	No	Yes	No
Feng, 2016^(41)^	Normal Lewis rat fetus	MSCs	Amniotic fluid and placenta (E21)	Sprague‐Dawley rat	Retinoic acid (60 mg/kg)	E10	1 × 10^5^ cells/uterine cavity	E17	Intra‐amniotic injection	E21	Yes	Yes	No
Shieh, 2018^(42)^	Normal Lewis rat fetus	MSCs	Amniotic fluid (E21)	Sprague‐Dawley rat	Retinoic acid (60 mg/kg)	E10	1 × 10^5^ cells/uterine cavity	E17	Intra‐amniotic injection	E22	Yes	No	No
Lazow, 2020^(43)^	Normal Lewis rat fetus	MSCs	Amniotic fluid (E21)	Sprague‐Dawley rat	Retinoic acid (60 mg/kg)	E10	1 × 10^5^ cells/uterine cavity	E17	Intra‐amniotic injection	E21	Yes	Yes	No
Shieh, 2019^(46)^	Fetal New Zealand rabbit	MSCs	Amniotic fluid	New Zealand rabbit	Surgical creation	E22–23	6 × 10^6^ cells/uterine cavity	E22–23; immediately after surgical creation	Intra‐amniotic injection	E30–32	Yes	Yes	No
Abe, 2019^(44)^	Human	MSCs	Amniotic fluid (GA 15–17 weeks)	Sprague‐Dawley rat	Retinoic acid (60 mg/kg)	E10	1 × 10^5^ cells/uterine cavity	E17	Intra‐amniotic injection	E21	Yes	Yes	Yes
Kajiwara, 2017^(45)^	Human, trisomy 21/TTTS	Skin derived from iPSCs	Amniotic fluid (GA 29 weeks)	Sprague‐Dawley rat	Retinoic acid (60 mg/kg)	E10	3D skin transplantation	E20	Collagen type I scaffold	E22	Yes	Yes	No
Fauza, 2008^(47)^	Mice	NSCs	Cerebellum	Ovine	Surgical creation	GA 97–112 days	2 × 10^8^ cells/spinal cord	14–25 days after surgical creation	Direct spinal cord injection	GA 145 days	Yes	No	Yes
Turner, 2013^(48)^	Lewis rat fetuses with NTDs	NSCs	Amniotic fluid (E19–E21)	Lewis rat	Retinoic acid (60 mg/kg)	E10	1.5 × 10^4^ cells/uterine cavity	E17	Intra‐amniotic injection	E21	Yes	No	No
Saadai, 2013^(49)^	Human	NCSCs	iPSCs	Ovine	Surgical creation	GA 75 days	2–3 x 10^7^ cells/spinal cord	GA 100 days	Hydrogel on nanofibrous scaffold* with	GA 135 days	Yes	No	No
Wang, 2015^(50)^	Human	MSCs	Placenta (11–17 weeks)	Ovine	Surgical creation	GA 77 days	5 × 10^5^ cells/spinal cord	GA 104 days	2 mg/ml rat tail collagen	GA 146 days	Yes	No	Yes
Brown, 2016^(51)^	Human	MSCs	Placenta (17 and 40 weeks)	Ovine	Surgical creation	GA 75 days	17 weeks; 5 × 10^5^ cells/spinal cord40 weeks; 1 × 10^6^ cells/spinal cord	GA 100 days; 25 days after surgical creation	2 mg/ml rat tail collagen	GA 145 days	Yes	No	Yes
Kabagambe, 2017^(52)^	Human	MSCs	Placenta (2nd trimester)	Ovine	Surgical creation	GA 78 days	5 × 10^5^ cells/spinal cord	GA 103 days; 25 days after surgical creation	SIS‐ECM	GA 145 days	No	No	Yes
Chen, 2017^(53)^	Human	MSCs	Placenta (15 weeks)	Sprague‐Dawley rat	Retinoic acid (40 mg/kg)	E10	1.6x, 3.1x, 6.3, 9.4 × 10^5^ cells/spinal cord	E19	SIS‐ECM	E21	No	No	Yes
Vanover, 2019^(54)^	Human	MSCs	Placenta (2nd trimester)	Ovine	Surgical creation	GA 77 days	5 × 10^5^, 2 × 10^6^ or 3 × 10^6^ cells/spinal cord	GA 102 days; 25 days after surgical creation	SIS‐ECM	GA 145 days	Yes	No	Yes
Galganski, 2019^(55)^	Human	MSCs	Placenta (14–21 weeks)	Ovine	Surgical creation	GA 76 days	3.6 × 10^6^ cells/spinal cord	GA 102 days	SIS‐ECM	GA 146 days	No	No	Yes

Abbreviations: BM, bone marrow; E, embryo; ESCs, embryonic stem cells; GA, gestational age in days using data from the study or calculated from study methods; HH, Hamburger and Hamilton stage;^62^ iPSCs, induced pluripotent stem cells; MMC, myelomeningocele; MSC, mesenchymal stem cells; NTDs, neural tube defects including exencephaly and/or myelomeningocele; NCSCs, neural crest stem cells; SIS‐ECM, small intestinal submucosa‐derived extracellular matrix; w, weeks of gestation; TTTS, twin to twin transfusion syndrome.

^a^ Volume ratio of NCSCs/hydrogel = 2:1 spread on nanofibrous scaffold comprising poly(L‐lactide‐co) caprolactone, polypropylene glycol and sodium acetate fabricated by electrospinning process.

### 3.4 Animal survival

**FIGURE 3:**
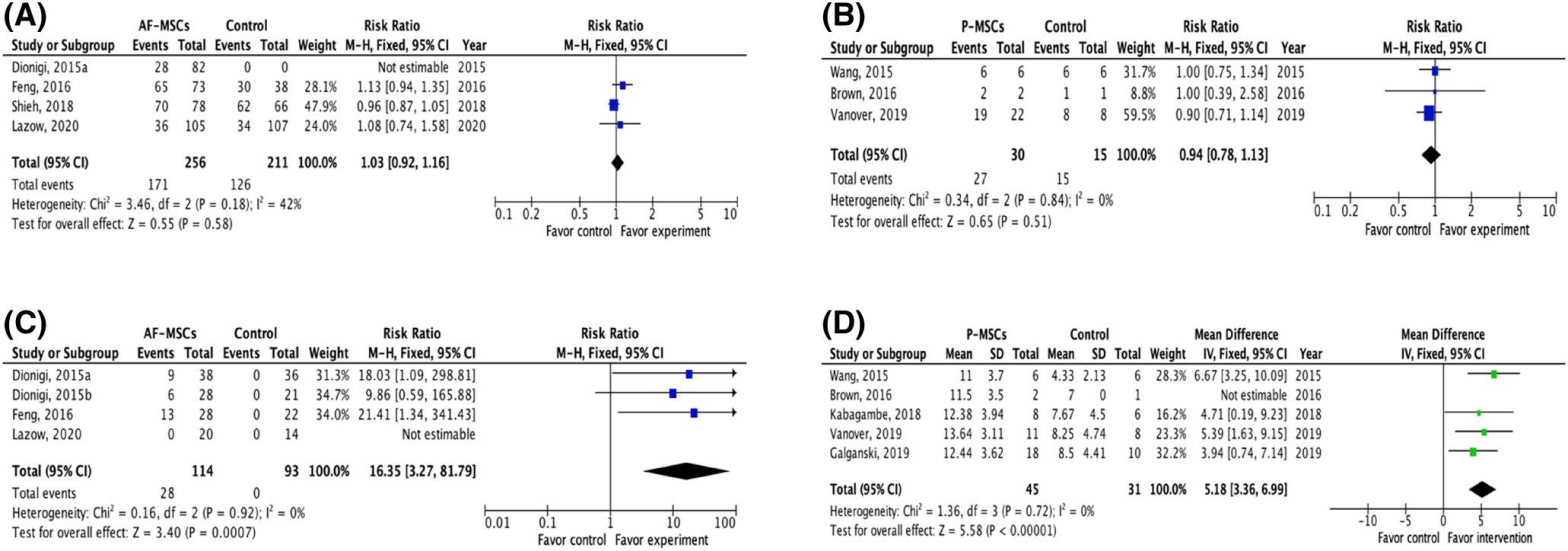
Meta‐analysis. (A) Meta‐analysis of fetal rat survival at term after intra‐amniotic injection of allogenic amniotic fluid‐derived mesenchymal stem cells or saline at E17.^37^, ^39^, ^40^, ^41^ Myelomeningocele (MMC) was created in all studies using retinoic acid. (B) Meta‐analysis of fetal lamb survival at term after application of human second trimester placental (P)‐mesenchymal stem cells (MSCs) during fetal surgical closure of MMC compared to fetal surgical closure alone.^48^, ^49^, ^52^ MMC was surgically created in these studies at Gestational Age (GA) 75–77 days; fetal surgical closure was performed 25 days later (GA 100–102 days). (C) Meta‐analysis of defect coverage in the retinoic acid‐induced fetal rat MMC model. Intra‐amniotic injection of allogenic amniotic fluid‐derived mesenchymal stem cells at E17 significantly increased the likelihood of total defect coverage compared to saline injection.^37^, ^38^, ^39^, ^41^ (D) Meta‐analysis of spinal cord function in the surgical fetal ovine model of MMC determined by sheep locomotor rating scale, after fetal surgery in conjunction with the application of human placental‐derived mesenchymal stem cells compared to fetal surgery alone^48^, ^49^, ^50^, ^52^, ^53^

Twenty‐one studies reported data on animal survival after in utero stem cell application,^30^, ^31^, ^32^, ^36^, ^37^, ^38^, ^39^, ^41^, ^42^, ^43^, ^44^, ^45^, ^46^, ^47^, ^48^, ^49^, ^50^, ^51^, ^54^ 13 (62%) of them presented data on survival rates in both control and treatment groups. Overall, there was no significant effect of stem cell application on animal survival rates (Table 2). Meta‐analysis was possible in four studies in the retinoic acid‐induced fetal rat MMC model^39^, ^41^, ^42^, ^43^ and three studies in a surgical ovine MMC model.^50^, ^51^, ^54^ The results showed that in the rat MMC model, when compared to saline injection, intra‐amniotic injection of allogeneic AF‐MSC at E17 of gestation, did not affect animal survival (RR 1.03, 95% CI 0.92–1.16; Figure 3A). Similarly, animal survival was not different in MMC sheep receiving application of human second trimester P‐MSCs to the spinal cord during fetal surgical closure of the MMC defect (compared to the control group undergoing fetal surgery alone (RR 0.94, 95% CI 0.78–1.13; Figure 3B).

**TABLE 2:** Animal survival after stem cell application

First author	Animal model/Stem cell	Survival rate, *n*/*N* (%)		
		Treatment	Control	*p* value
Lee, 2004^(30)^	Chicken embryo/Human ESCs	POD 3, NR	POD 4, NR	>0.05
		POD 5, NR	POD 6, NR	>0.05
		POD 7, NR	POD 8, NR	<0.05
Lee, 2006^(31)^	Chicken embryo/Human ESCs	POD 4, 15/19 (78.9%)	POD 4, 15/18 (83.3%)	0.73
		POD 6, 15/21 (71.4%)	POD 6, 15/19 (78.9%)	0.58
		POD 8, 15/23 (65.2%)	POD 8, 15/22 (68.2%)	0.83
Li, 2012^(33)^	Wistar rat/Wistar rat BM‐MSCs	152/195 (77.9%)	NA	NA
Li, 2014^(34)^	Wistar rat/Wistar rat BM‐MSCs	18/22 (81.8%)	NA	NA
Ma, 2015^(35)^	Wistar rat/Wistar rat BM‐MSCs	58/72 (80.6%)	NA	NA
Li, 2016^(36)^	Wistar rat/Wistar rat BM‐MSCs	69/134 (51.5%)	NA	NA
Wei, 2020a^(37)^	Wistar rat/Wistar rat BM‐MSCs	30/30 (100%)	23/23 (100%)	1.00
Wei, 2020b^(38)^	Wistar rat/Wistar rat BM‐MSCs	32/32 (100%)	28/28 (100%)	1.00
Turner, 2013^(48)^	Lewis rat/Lewis rat AF‐NSCs	20/37 (54.1%)	NA	NA
Dionigi, 2015a^(39)^	Sprague‐Dawley rat/Lewis rat AF‐MSCs	28/82 (34.1%)	NA	NA
Feng, 2016^(41)^	Sprague‐Dawley rat/Lewis rat AF‐MSCs and P‐MSCs	**AF‐MSCs**, 65/73 (89.0%)	30/38 (78.9%)	0.15
		**P‐MSCs**, 90/115 (78.3%)		0.93
Shieh, 2018^(42)^	Sprague‐Dawley rat/Lewis rat AF‐MSCs	70/78 (89.7%)	62/66 (93.9%)	0.77
Lazow, 2020^(43)^	Sprague‐Dawley rat/Lewis rat AF‐MSCs	36/105 (34.3%)	34/107 (31.8%)	0.70
Abe, 2019^(44)^	Sprague‐Dawley rat/Human AF‐MSCs	19/22 (86.4%)	17/19 (89.5%)	0.76
Kajiwara, 2017^(45)^	Sprague‐Dawley rat/3D skin from human AF‐derived iPSCs	20/20 (100%)	NA	NA
Shieh, 2019^(46)^	New Zealand rabbit/New Zealand rabbit AF‐MSCs	10/35 (28.6%)	5/15 (33.3%)	0.74
Fauza, 2008^(47)^	Ovine/Mice cerebellum NSCs	8/9 (88.8%)	6/7 (85.7%)^a^	0.85
Saadai, 2013^(49)^	Ovine/Human NCSCs derived from iPSCs	2/2 (100%)	NA	NA
Wang, 2015^(50)^	Ovine/Human P‐MSCs	6/6 (100%)	6/6 (100%)^a^	1.00
Brown, 2016^(51)^	Ovine/Human P‐MSCs	2/2 (100%)	1/1 (100%)^a^	1.00
Vanover, 2019^(54)^	Ovine/Human P‐MSCs	19/22 (86.4%)	8/8 (100%)^a^	0.55

Abbreviations: AF‐MSCs, amniotic fluid‐derived mesenchymal stem cells; CRL, crown‐rump length; NA, not available; NR, exact data are not retrievable after contact with corresponding author; P‐MSCs, placental‐derived mesenchymal stem cells; POD, postoperative day.

^a^ Fetal MMC surgical repair as a control group.

## 4 EFFICACY OF TREATMENT

### 4.1 Coverage of the MMC defect

Outcomes following defect coverage were reported in 13 studies.^30^, ^31^, ^32^, ^36^, ^37^, ^38^, ^39^, ^40^, ^41^, ^43^, ^44^, ^45^, ^46^ The coverage was evaluated in a number of ways (Table 3) such as gross complete defect coverage with microscopic confirmation, absolute defect area at birth, and adjusted defect length to original incision length and body length. The most commonly used stem cells for this purpose were MSCs (76.9%, 10/13); almost half of the studies were human xenogeneic transplantation (38.5%, 5/13). Studies were conducted exclusively in small/medium‐size animal models; 69.2% (9/13) in rat species, 23.1% (3/13) in chicken embryos and 7.7% (1/13) in rabbit species. Outcomes of defect coverage are summarized in Table 3.

**TABLE 3:** Effects of stem cell transplantation on coverage of the defect

First author	Stem cell/animal model	Treatment group	Control group	Evaluation method	Effect and time of evaluation		
					Treatment Group	Control Group	*p* value
Lee, 2004^(30)^	Human ESCs/Chicken embryo	Intra‐amniotic injection of ESCs^a^	Intra‐amniotic injection of glucose in PBS (4.5 mg/L)^a^	Adjusted defect length (defect length/original incision length × total body length)	**POD 3,** 0.03	**POD 3,** 0.07	<0.01
					**POD 5,** 0.02	**POD 5,** 0.07	<0.01
					**POD 7,** 0.02	**POD 7,** 0.08	<0.01
Lee, 2006^(31)^	Human ESCs/Chicken embryo	Intra‐amniotic injection of ESCs (*n* = 15 at all time points)	No intra‐amniotic injection (*n* = 15 at all‐time points)	Adjusted defect length (defect length/original incision length × total body length)	**POD 4,** 0.05	**POD 4,** 0.08	<0.01
					**POD 6,** 0.03	**POD 6,** 0.07	<0.01
					**POD 8,** 0.04	**POD 8,** 0.08	<0.01
Lee, 2010^(32)^	Human NCSCs and BM‐MSCs/Chicken embryo	Intra‐amniotic injection of NSCs or BM‐MSCs^a^	No intra‐amniotic injection^a^	Adjusted defect length (defect length/original incision length × total body length)	**BM‐MSCs, POD 3,** 0.05	**POD 3,** 0.08	<0.01
					**BM‐MSCs, POD 5,** 0.05	**POD 5,** 0.08	<0.01
					**BM‐MSCs, POD 7,** 0.05	**POD 7,** 0.08	<0.01
					**NSCs, POD 3,** 0.08		ns
					**NSCs, POD 5,** 0.07		ns
					**NSCs, POD 7,** 0.08		ns
Li, 2016^(36)^	Wistar rat BM‐MSCs/Wistar rat	BM‐MSCs seeded on SIS‐ECM	No surgery	No. of animals with complete defect coverage evaluated macroscopically, n/N (%)	47/69 (68.1%)	0/30 (0%)	<0.01
Wei, 2020a^(37)^	Wistar rat BM‐MSCs/Wistar rat	Intra‐amniotic injection BM‐MSCs (*n* = 32)	Intra‐amniotic injection of PBS (*n* = 28)	Absolute defect area (mm^2^)	57.4 ± 4.1 mm^2^	80.2 ± 4.8 mm^2^	<0.01
Wei, 2020b^(38)^	Wistar rat/Wistar rat BM‐MSCs	Intra‐amniotic injection BM‐MSCs (*n* = 30)	Intra‐amniotic injection of PBS (*n* = 23)	Absolute defect area (mm^2^)	78.3 ± 6.3 mm^2^	54.9 ± 4.6 mm^2^	<0.05
Dionigi, 2015a^(39)^	Lewis rat AF‐MSCs/Sprague‐Dawley rats	Intra‐amniotic injection of AF‐MSCs	Intra‐amniotic injection of PBS	No. of animals with defect coverage evaluated macroscopically with microscopic confirmation, n/N (%)	31/38 (81.6%) complete coverage, 9/38 (23.7%)	0/36 (0%)	<0.01
Dionigi, 2015b^(40)^	Lewis rat AF‐MSCs/Sprague‐Dawley rats	Intra‐amniotic injection of AF‐MSCs	No injection	No. of animals with defect coverage evaluated macroscopically with microscopic confirmation, n/N (%)	24/28 (85.7%) complete coverage, 6/28 (21.4%)	0/21 (0%)	<0.01
Feng, 2016^(41)^	Lewis rat AF‐MSCs and P‐MSCs/Sprague‐Dawley rats	Intra‐amniotic injection of AF‐MSCs	Intra‐amniotic injection of PBS	No. of animals with defect coverage evaluated macroscopically with microscopic confirmation, n/N (%)	**AF‐MSCs**, 13/28 (46.4%) complete coverage, 3/28 (10.7%)	0/22 (0%)	<0.01
					**P‐MSCs**, 18/38 (47.4%), complete coverage, 2/38 (5.3%)		
Lazow, 2020^(43)^	Lewis rat AF‐MSCs and P‐MSCs/Sprague‐Dawley rats	Intra‐amniotic injection of AF‐MSCs	Intra‐amniotic injection of PBS	No. of animals with defect coverage evaluated macroscopically with microscopic confirmation, n/N (%)	11/20 (55.0%) complete coverage; 0/20 (0%)	0/14 (0%)	<0.01
				Ratio of defect area/total area of the head and back (dorsal surface)	Median: 0.09, IQR: 0.06–0.16	Median: 0.06, IQR: 0.04–0.09	<0.01
Shieh, 2019^(46)^	New Zealand rabbit AF‐MSCs/New Zealand rabbit	Intra‐amniotic injection of AF‐MSCs	No intra‐amniotic injection	No. of animals with defect coverage evaluated macroscopically with microscopic confirmation, n/N (%)	5/10 (50.0%) complete coverage; 0/10 (0%)	0/5 (0%)	<0.01
Abe, 2019^(44)^	Human AF‐MSCs/Sprague‐Dawley rat	Intra‐amniotic injection of AF‐MSCs (*n* = 19)	Intra‐amniotic injection of PBS (*n* = 17)	Absolute defect area and adjusted defect area (area/CRL^2^)	**Area,** 53.9 ± 11.8 mm^2^	**Area,** 39.2 ± 8.1 mm^2^	0.01
					**Adjusted area,** 0.02 ± 0.004	**Adjusted area,** 0.03 ± 0.009	0.03
Kajiwara, 2017^(45)^	3D skin from human AF‐derived iPSCs/Sprague‐Dawley rat	3D skin surgical application	No intra‐amniotic procedure	No. of animals with defect coverage evaluated macroscopically with microscopic confirmation, n/N (%)	12/20 (60.0%) complete coverage, 4/12 (33.3%)	0/61 (0%)	<0.01

*Note*: Data presented with mean ± SEM.

Abbreviations*:* PBS, phosphate‐buffered saline; CRL, crown‐rump length; ESCs, embryonic stem cells; FM, fetal membranes; iPSCs, induced pluripotent stem cells; MSC, mesenchymal stem cells; NCSCs, neural crest stem cells.

^a^ Number of animals not provided.

Meta‐analysis of defect coverage was possible in four studies in the retinoic acid‐induced fetal rat MMC model where there was allogeneic intra‐amniotic injection of AF‐MSCs from normal rat fetuses at E17. Stem cell injection was associated with a higher likelihood of complete defect coverage when compared to control saline injection (RR 16.35, 95% CI 3.2–81.79)^39^, ^40^, ^41^ (Figure 3C). One further study comparing the application of placental MSCs (P‐MSCs) to that of AF‐MSCs in the same retinoic acid‐induced fetal rat MMC model at the same stage of gestation, demonstrated that there was no difference in defect coverage (complete coverage; AF‐MSC 10.7% vs. P‐MSCs 5.3%, *p* = 0.41).^41^ In the surgically created rabbit model of MMC, intra‐amniotic injection of allogeneic AF‐MSCs on the day of MMC surgical creation (E22‐23 days) significantly increased the likelihood of defect coverage, with 50% of the animals showing some degree of defect coverage; however, none had complete coverage.^46^

In terms of human xenogeneic transplantation, one study found that intra‐amniotic injection of human AF‐MSCs in the retinoic acid‐induced fetal rat MMC model at E17 significantly reduced the area of the MMC defect compared to saline injection (Table 3).^44^ Another study demonstrated that in utero transplantation of 3‐dimensional (3D) skin generated from human AF‐derived iPSCs resulted in more rats having some degree of defect coverage compared to no transplantation (Table 3).^45^

### 4.2 Spinal cord histopathology and function

There were 11 studies reporting the effect of stem cells on spinal cord histopathology and/or function with almost all using MSCs (90.1%, 10/11); 63.6% (7/11) of studies applied human MSCs.^34^, ^35^, ^38^, ^44^, ^47^, ^50^, ^51^, ^52^, ^53^, ^54^, ^55^ Fetal surgical ovine and retinoic acid‐induced fetal rat models of MMC were used in 54.5% (6/11) and 45.5% (5/11) of the studies, respectively. Improvement of spinal cord outcomes are shown in Table 4. Meta‐analysis to study the spinal cord function was possible in five studies in the surgically created ovine model of MMC (Figure 3D). Incorporation of P‐MSCs at the time of MMC fetal surgical closure improved motor function of the lower limbs compared to fetal surgery alone, as determined by SLR scale (mean difference 5.18, 95% CI 3.36–6.99; Figure 3D). The density of large neurons was also found to be increased with the intervention (Table 4).

**TABLE 4:** Effects of stem cell transplantation on spinal cord histopathology and/or function

First author	Stem cell/animal model	Treatment group	Control group	Histology analysis				Functional analysis			
				Method	Treatment	Control	*p* value	Method	Treatment	Control	*p* value
Li, 2014^(34)^	Wistar rat BM‐MSCs/Wistar rat	Spinal cord injection of BM‐MSCs	No injection	TUNEL analysis (% of death cells/total cells)	4.8 ± 0.3	8.9 ± 0.6	<0.05	NA	NA	NA	NA
Ma, 2015^(35)^	Wistar rat BM‐MSCs/Wistar rat	Spinal cord injection of BM‐MSCs (*n* = 15)	No injection (*n* = 10)	Sensory neuron in dorsal root ganglion (Brn3a + ve cells/total cells)	33.4 ± 1.9%	25.3% ± 1.6%	<0.01	NA	NA	NA	NA
Wei, 2020b^(38)^	Wistar rat BM‐MSCs/Wistar rat	Intra‐amniotic injection BM‐MSCs (*n* = 12)	Intra‐amniotic injection of PBS (*n* = 12)	NA	NA	NA	NA	Motor evoked potential of anterior tibialis muscle (millivolts)	0.26 ± 0.02	0.18 ± 0.02	<0.05
								Latency period of electrophysiologic test between cortex and anterior tibialis muscle (milliseconds)	22.8 ± 0.3	25.4 ± 0.8	<0.05
Abe, 2019^(44)^	Human AF‐MSCs/Sprague‐Dawley rat	Intra‐amniotic injection of AF‐MSCs (*n* = 19)	Intra‐amniotic injection of PBS (*n* = 17)	Cross‐sectional area of spinal cord (mm^2^)	1.9 ± 0.2	0.9 ± 0.1	0.01	NA	NA	NA	NA
				Ratio of GFAP/βIII‐tubulin cross‐sectional area	0.3 ± 0.1	0.7 ± 0.2	<0.05				
Chen, 2017^(53)^	Human P‐MSCs/Sprague‐Dawley rat	P‐MSCs seeded on SIS‐ECM	SIS‐ECM and surgical closure (*n* = 9)	Cross‐sectional ratio of spinal cord width/height	Group 1, 2.9 ± 0.5	5.5 ± 0.7	<0.01	NA	NA	NA	NA
		Group 1, 1.6 × 10^5^ cells/spinal cord, *n* = 12			Group 2, 3.2 ± 0.5		0.02				
		Group 2, 3.1 × 10^5^ cells/spinal cord, *n* = 6			Group 3, 3.3 ± 0.4		0.01				
		Group 3, 6.3 × 10^5^ cells/spinal cord, *n* = 9			Group 4, 3.2 ± 0.3		<0.01				
		Group 4, 9.4 × 10^5^ cells/spinal cord, *n* = 10		Density of apoptotic cells (cells/mm^3^)	Group 1, 74.8 ± 30.2	200.5 ± 54.57	ns				
					Group 2, 116.4 ± 59.5		ns				
					Group 3, 50.6 ± 23.3		ns				
					Group 4, 49.2 ± 7.3		0.04				
Fauza, 2008^(47)^	Mice cerebellum NSCCs/Ovine	Spinal cord injection of NCSCs and surgical closure with AlloDerm (*n* = 6)	Surgical closure with AlloDerm (*n* = 8)	NA	NA	NA	NA	Ability to walk	2/6 (33.3%)	2/8 (25%)	0.73
Wang, 2015^(50)^	Human P‐MSCs/Ovine	P‐MSCs mixed with rat tail collagen and surgical closure with Oasis patch (*n* = 6)	Rat tail collagen and surgical closure with Oasis patch (*n* = 6)	Large neuron density^a^	NR	NR	0.01	SLR scale (median, range)	11.5 (5–15)	4 (2–8)	0.01
		5 × 10^5^ cells/spinal cord									
Brown, 2016^(51)^	Human P‐MSCs/Ovine	17 weeks‐P‐MSCs (*n* = 1) or 40 weeks‐P‐MSCs (*n* = 1) with fetal membranes and surgical closure	Fetal membranes with surgical closure (*n* = 1)	Cross‐sectional area of spinal cord (mm^2^)	**17 weeks,** 5.6	5.6	NA	SLR scale	**17 weeks,** 15**40–week**, 8	7	NA
					**40 weeks,** 8.7						
				Cross‐sectional area of grey matter (mm^2^)	**17 weeks,** 1.3	1.8	NA				
					**40 weeks,** 6.9						
				Large neuron density^a^ (cell/mm^2^)	**17 weeks,** 12.2	5.0	NA				
					**40 weeks,** 10.7						
Kabagambe, 2017^(52)^	Human P‐MSCs/Ovine	P‐MSCs seeded on SIS‐ECM and surgical closure (*n* = 8)	SIS‐ECM and surgical closure (*n* = 6)	Cross‐sectional area of spinal cord (mm^2^)	8.4 ± 1.8	7.4 ± 2.0	0.57	SLR scale (median, range)	15 (4–15)	6 (3–15)	0.09
		5 × 10^5^ cells/spinal cord		Cross‐sectional area of grey matter (mm^2^)	2.0 ± 0.6	1.4 ± 0.5	0.57				
				Large neuron density^a^	18.8 ± 4.3	13.9 ± 7.0	0.23				
Vanover, 2019^(54)^	Human P‐MSCs/Ovine	P‐MSCs seeded on SIS‐ECM and surgical closure	SIS‐ECM and surgical closure (*n* = 8)	Normalized cross‐sectional area of spinal cord (mm^2^)^b^	Group 1, 0.4 ± 0.3	0.5 ± 0.3	ns	SLR scale (median, range)	Group 1, 15 (4–15)	6.5 (3–15)	0.04
		Group 1, 5 × 10^5^ cells/spinal cord, *n* = 8			Group 2, 0.9 ± 0.1		<0.05		Group 2, 15 (4–15)		
		Group 2, 2 × 10^6^ cells/spinal cord, *n* = 6			Group 3, 0.7 ± 0.2		ns		Group 3, 15 (14,15)		
		Group 3, 3 × 10^6^ cells/spinal cord, *n* = 5		Normalized cross‐sectional area of grey matter (mm^2^)^b^	Group 1, 0.4 ± 0.3	0.4 ± 0.3	ns				
					Group 2, 0.9 ± 0.3		<0.05 ns				
					Group 3, 0.6 ± 0.3						
				Normalized large neuron density (cells/mm^3^)^a^ ^,^ ^b^	Group 1, 0.8 ± 0.5	0.5 ± 0.4	ns				
					Group 2, 0.8 ± 0.3		ns				
					Group 3, 1.0 ± 0.3		<0.05				
Galganski, 2019^(55)^	Human P‐MSCs/Ovine	P‐MSCs (line A, *n* = 6, line B, *n* = 7, line C, *n* = 5) seeded on SIS‐ECM and surgical closure	SIS‐ECM and surgical closure (*n* = 10)	Large neuron density (cells/mm^3^)^a^ ^,^ ^c^	Line A, 25.2 (19.1–30.4),	4.7 (2.7–13.7)	0.04	SLR scale (median, range)	Line A, 15 (8–15)	7.5 (3–15)	
		3.6 × 10^6^ cells/spinal cord			Line B, 27.6 (3.4–33.2)		0.04		Line B, 14 (7–15)		
					Line C, 24.8 (12.3–28.1)		ns		Line C, 14 (14–15)		

*Notes*: Data presented with mean ± SEM. Rat tail collagen (BD Biosciences), Oasis patch (Cook Biotech), AlloDerm (LifeCell), SIS‐ECM (Cook Biotech).

Abbreviations: ESCs, embryonic stem cells; iPSCs, induced pluripotent stem cells; FM, fetal membranes; MSC, mesenchymal stem cells; NA, not available; NCSCs, neural crest stem cells; NR, exact data are not retrievable after contact with corresponding author; SLR, sheep locomotor rating scale (highest score = 15)^63^; SIS‐ECM, small intestinal submucosa‐derived extracellular matrix.

^a^ Large neuron density = number of 30–70 μm diameter‐neurons/cross‐sectional area of grey matter.

^b^ Normalised to average cross‐sectional area of corresponding lumbar level of normal newborn ovines.

^c^ Data presented with median (interquartile range).

In small animal models, injection of adult rat BM‐MSCs at E16 into the spinal cord of retinoic acid generated fetal rats with MMC, was associated with a reduction in spinal cord cell death assessed by TUNEL analysis at E20 (death cells; 4.8 ± 0.3% vs. 8.9 ± 0.6%, *p* < 0.05),^34^ and an increase in the number of sensory neurons in the dorsal root ganglion (33.4 ± 1.9% vs. 25.3 ± 1.6%, *p* < 0.01).^35^ The intervention also improved corticospinal tract communication to the anterior tibialis muscle, demonstrated by a rise in motor evoked potentials (0.26 ± 0.02 mV vs. 0.18 ± 0.02 mV, *p* < 0.05) and a shorter latency period (22.8 ± 0.3 ms vs. 25.4 ± 0.8 ms, *p* < 0.05).^38^

One study has studied the direct injection of mouse‐derived NCSCs into the spinal cord of fetal lambs with surgically created MMC at approximately gestational day 125 did not improve limb motor function after birth (2/6%, 33% vs. 2/8%, 25%, *p* = 0.73).^47^ Although the cells did not differentiate, xenogeneic cells were able to engraft and produce the neurotrophic factors glial cell line‐derived neurotrophic factor and brain‐derived neurotrophic factor.^47^ Another study demonstrated that human xenogeneic NCSCs delivered to fetal ovine spinal cord via nanofibrous scaffold survived and integrated with host neurons. These cells made up 35%‐70% of neurons in the examined area.^49^

## 5 DISCUSSION

This systematic review summarizes 26 studies in a narrative synthesis and nine studies by meta‐analysis in the evaluation of the safety and efficacy of stem cell transplantation in animal models of MMC. We found that a variety of stem cells types, delivery techniques and animal models had been used. Overall the results suggest beneficial benefits of stem cells on animal survival, defect coverage and spinal cord function. Safety data represented by animal survival rates were reassuring; both for intra‐amniotic injection of allogeneic AF‐MSCs in the fetal rat model and the application of P‐MSCs to the spinal cord during fetal surgical MMC closure in the MMC lamb model did not compromise fetal survival at term. In terms of efficacy, a single injection of allogeneic AF‐MSCs into the intra‐amniotic cavity of fetal rats was associated with a higher rate of complete defect coverage compared to injection of saline. In addition, the incorporation of human P‐MSCs as a therapeutic adjunct to fetal surgical MMC closure in the ovine model, when compared with fetal surgery alone, significantly improved the motor function of the newborn lambs.

Current clinical fetal surgery approaches are highly invasive and may come (too) late for (full) recovery. This is the rationale for less invasive approaches, such as intra‐amniotic injection of stem cells, to assist in defect coverage early in gestation. In addition, this approach may complement several shortcomings of fetal surgery as not all MMC fetuses are eligible for fetal surgery and not all fetal centres offer this service. The concept of intra‐amniotic injection of allogeneic AF‐MSCs from normal fetuses to induce MMC defect coverage has been shown efficacious in the fetal rat MMC model.^39^, ^40^, ^41^, ^43^ In most of the included studies, the coverage occurred by means of rudimentary skin development. The mechanism behind this may resemble how MSCs improve cutaneous wound healing via their differentiation and paracrine effects, which are vital in all stages of the healing process.^56^, ^57^ Although in the rat model, complete coverage occurred in almost one third of the animals with a single injection of AF‐MSCs, none was documented in the larger rabbit model and there was no data regarding neurological improvements. In light of this, the efficacy of intra‐amniotic AF‐MSCs to induce defect coverage and eventually to improve neurological functions remains to be evaluated in both small and large animal models. This is important if we consider that, in rodents and rabbits, the volume of intra‐amniotic cavity and the gestation are respectively smaller and relatively shorter than in the ovine and/or eventually the human. The use of large animal models will provide further information that can be translated in future clinical trials; for example, the technique for stem cell delivery, the determination of appropriate stem cell dosage and the number of injections required to achieve a complete defect coverage.^58^ As the intra‐amniotic volume of humans is much larger than that of the rat, improvements in a technique or vehicle to deliver stems needs further development in order to promote cell survival, migration and attachment. The longer gestational period in large animal models would also allow information on medium‐to‐long term effects of MSCs such as cell engraftment and characterisation of regenerated skin layers.

The rationale for incorporating stem cells as an adjunct to fetal surgery is to regenerate the ‘already damaged’ spinal cord as even after fetal surgery, more than half of children with MMC were unable to walk without orthoses.^11^ In this systematic review, we found a significant improvement in motor function of the lower limbs in newborn lambs receiving P‐MSCs during fetal surgical closure of the MMC defect. Recovery of spinal cord function by MSC therapy is supported by evidence from a recent clinical meta‐analysis in adults suffering from spinal cord injury. The study showed that subarachnoid or intravenous injection of MSCs into those patients, improved the overall spinal cord injury scale, sensory and bladder functions when compared with rehabilitation therapy alone.^22^ It is postulated that MSCs rescue neural regeneration via their paracrine effects. In fetal MMC animal models, the cells were shown to modulate the neuroinflammatory response, exert neurotrophic effects and promote angiogenesis through the secretion of growth factors, cytokines and extracellular vesicles.^59^, ^60^

Although our findings are encouraging for clinical translation, further work is needed to determine the optimal source and dose of P‐MSCs with appropriate toxicology studies before moving to a phase 1 clinical trial of P‐MSCs as an adjunct to fetal surgery. Using autologous AF‐MSCs for clinical treatment is also a feasible option as the majority of MMC fetuses are diagnosed in the second trimester, and women who wish to proceed to fetal surgery are mandated to undergo an amniocentesis to determine fetal karyotype.^5^ Hence, amniotic fluid would be available for MSC isolation in most cases. A recent review provides more details about the experiments in each study and comes to a similar conclusion.^61^

Our systematic review is limited for two reasons. First, we only included studies published in English language which may omit eligible studies reported in other languages. Second, studies included in this review carry a high risk of bias due to lack of detail on randomization, allocation and treatment concealment and lastly selective outcome reporting. Although, the majority of included studies considered animal baseline characteristics, very few explicitly described the method of randomization and/or concealment applied in their studies. Furthermore, none of the studies provide adequate information on animal housing and further care. For this reason, we encourage authors to enhance the quality of their scientific reports by following the guidance of the ARRIVE guidelines.^29^ Ultimately, as with all translational research, there is an inevitable risk that the benefits of stem cell application would be overestimated owing to publication bias.

## 6 CONCLUSIONS

Intra‐amniotic injection of allogeneic AF‐MSCs is safe and effective in inducing MMC defect coverage in small animal models; however, there are no data in large animal models. Transplantation of human P‐MSCs to the spinal cord of fetal lambs with MMC, as an adjunct to fetal surgery, is also safe and effective in enhancing lower limb motor function of newborn lambs after delivery.

Although our findings are encouraging for clinical translation, there are several concerns that needed to be addressed. Further work on neurological functional outcomes (beyond 24 h) after birth and the response of the fetal immune system to allogeneic stem cell transplantation should also be taken into consideration. Apart from that, an optimum stem cell source and an appropriate delivery device should be established before moving forward to clinical trial.

## CONFLICT OF INTEREST

The authors declare that there is no conflict of interest.

## Supporting information

Supplementary Material

Supplementary Material

## Data Availability

The data that supports the findings of this study are available in the supplementary material of this article.

## References

[1] Greene ND , Copp AJ . Neural tube defects. Annu Rev Neurosci. 2014;37:221‐242. 25032496 10.1146/annurev-neuro-062012-170354 PMC4486472

[2] Zaganjor I , Sekkarie A , Tsang BL , et al. Describing the prevalence of neural tube defects worldwide: a systematic literature review. PLoS One. 2016;11(4):e0151586. 27064786 10.1371/journal.pone.0151586 PMC4827875

[3] Syngelaki A , Hammami A , Bower S , Zidere V , Akolekar R , Nicolaides KH . Diagnosis of fetal non‐chromosomal abnormalities on routine ultrasound examination at 11‐13 weeks' gestation. Ultrasound Obstet Gynecol. 2019;54(4):468‐476. 31408229 10.1002/uog.20844

[4] Ovaere C , Eggink A , Richter J , et al. Prenatal diagnosis and patient preferences in patients with neural tube defects around the advent of fetal surgery in Belgium and Holland. Fetal Diagn Ther. 2015;37(3):226‐234. 25301576 10.1159/000365214

[5] Sacco A , Ushakov F , Thompson D , et al. Fetal surgery for open spina bifida. Obstet Gynaecol. 2019;21(4):271‐282. 31787844 10.1111/tog.12603 PMC6876677

[6] Sival DA , Begeer JH , Staal‐Schreinemachers AL , Vos‐Niel JM , Beekhuis JR , Prechtl HF . Perinatal motor behaviour and neurological outcome in spina bifida aperta. Early Hum Dev. 1997;50(1):27‐37. 9467691 10.1016/s0378-3782(97)00090-x

[7] Meuli M , Meuli‐Simmen C , Yingling CD , et al. Creation of myelomeningocele in utero: a model of functional damage from spinal cord exposure in fetal sheep. J Pediatr Surg. 1995;30(7):1028‐1032. 7472926 10.1016/0022-3468(95)90335-6

[8] Ben Miled S , Loeuillet L , Duong Van Huyen JP , et al. Severe and progressive neuronal loss in myelomeningocele begins before 16 weeks of pregnancy. Am J Obstet Gynecol. 2020;223(2):256.e1‐256.e9. 32283072 10.1016/j.ajog.2020.02.052

[9] Adzick NS , Thom EA , Spong CY , et al. A randomized trial of prenatal versus postnatal repair of myelomeningocele. N Engl J Med. 2011;364(11):993‐1004. 21306277 10.1056/NEJMoa1014379 PMC3770179

[10] Tulipan N , Wellons JC, 3rd , Thom EA , et al. Prenatal surgery for myelomeningocele and the need for cerebrospinal fluid shunt placement. J Neurosurg Pediatr. 2015;16(6):613‐620. 26369371 10.3171/2015.7.PEDS15336 PMC5206797

[11] Farmer DL , Thom EA , Brock JW, 3rd , et al. The Management of Myelomeningocele Study: full cohort 30‐month pediatric outcomes. Am J Obstet Gynecol. 2018;218(2):256.e1‐256.e13. 29246577 10.1016/j.ajog.2017.12.001 PMC7737375

[12] Brock JW, 3rd , Carr MC , Adzick NS , et al. Bladder function after fetal surgery for myelomeningocele. Pediatrics. 2015;136(4):e906‐e913. 26416930 10.1542/peds.2015-2114 PMC4586733

[13] Brock JW, 3rd , Thomas JC , Baskin LS , et al. Effect of prenatal repair of myelomeningocele on urological outcomes at school age. J Urol. 2019;202(4):812‐818. 31075056 10.1097/JU.0000000000000334

[14] Horst M , Mazzone L , Schraner T , et al. Prenatal myelomeningocele repair: do bladders better? Neurourol Urodyn. 2017;36(6):1651‐1658. 27862250 10.1002/nau.23174

[15] Pastuszka A , Bohosiewicz J , Koszutski T . Prenatal myelomeningocele repair improves urinary continence and reduces the risk of constipation. Neurourol Urodyn. 2018;37(8):2792‐2798. 30058735 10.1002/nau.23771

[16] Macedo A, Jr , Ottoni SL , Garrone G , et al. In utero myelomeningocoele repair and urological outcomes: the first 100 cases of a prospective analysis. Is there an improvement in bladder function? BJU Int. 2019;123(4):676‐681. 30548158 10.1111/bju.14639

[17] Sacco A , Simpson L , Deprest J , David AL . A study to assess global availability of fetal surgery for myelomeningocele. Prenat Diagn. 2018;38(13):1020‐1027. 30378145 10.1002/pd.5383 PMC7613409

[18] Adzick NS . Fetal surgery for spina bifida: past, present, future. Semin Pediatr Surg. 2013;22(1):10‐17. 23395140 10.1053/j.sempedsurg.2012.10.003 PMC6225063

[19] Soni S , Moldenhauer JS , Spinner SS , et al. Chorioamniotic membrane separation and preterm premature rupture of membranes complicating in utero myelomeningocele repair. Am J Obstet Gynecol. 2016;214(5):647.e1‐647.e7. 26692177 10.1016/j.ajog.2015.12.003

[20] Wyatt LA , Keirstead HS . Stem cell‐based treatments for spinal cord injury. Prog Brain Res. 2012;201:233‐252. 23186718 10.1016/B978-0-444-59544-7.00012-3

[21] Cho SR , Kim YR , Kang HS , et al. Functional recovery after the transplantation of neurally differentiated mesenchymal stem cells derived from bone marrow in a rat model of spinal cord injury. Cell Transpl 2016;25(7):1423. 10.3727/096368916X692078 28836830

[22] Xu P , Yang X . The efficacy and safety of mesenchymal stem cell transplantation for spinal cord injury patients: a meta‐analysis and systematic review. Cell Transpl 2019;28(1):36‐46. 10.1177/0963689718808471 PMC6322141 30362373

[23] George TM , Fuh E . Review of animal models of surgically induced spinal neural tube defects: implications for fetal surgery. Pediatr Neurosurg. 2003;39(2):81‐90. 12845198 10.1159/000071319

[24] Joyeux L , Engels AC , Van Der Merwe J , et al. Validation of the fetal lamb model of spina bifida. Sci Rep. 2019;9(1):9327. 31249378 10.1038/s41598-019-45819-3 PMC6597719

[25] Danzer E , Schwarz U , Wehrli S , Radu A , Adzick NS , Flake AW . Retinoic acid induced myelomeningocele in fetal rats: characterization by histopathological analysis and magnetic resonance imaging. Exp Neurol. 2005;194(2):467‐475. 15893307 10.1016/j.expneurol.2005.03.011

[26] Moher D , Liberati A , Tetzlaff J , Altman DG , Group P . Preferred reporting items for systematic reviews and meta‐analyses: the PRISMA statement. BMJ. 2009;339:b2535. 19622551 10.1136/bmj.b2535 PMC2714657

[27] Hooijmans CR , Rovers MM , de Vries RB , Leenaars M , Ritskes‐Hoitinga M , Langendam MW . SYRCLE's risk of bias tool for animal studies. BMC Med Res Methodol. 2014;14:43. 24667063 10.1186/1471-2288-14-43 PMC4230647

[28] Higgins JP , Thompson SG , Deeks JJ , Altman DG . Measuring inconsistency in meta‐analyses. BMJ. 2003;327(7414):557‐560. 12958120 10.1136/bmj.327.7414.557 PMC192859

[29] Kilkenny C , Browne W , Cuthill IC , Emerson M , Altman DG , Group NCRRGW . Animal research: reporting in vivo experiments: the ARRIVE guidelines. Br J Pharmacol. 2010;160(7):1577‐1579. 20649561 10.1111/j.1476-5381.2010.00872.x PMC2936830

[30] Lee DH , Park S , Kim EY , et al. Enhancement of re‐closure capacity by the intra‐amniotic injection of human embryonic stem cells in surgically induced spinal open neural tube defects in chick embryos. Neurosci Lett. 2004;364(2):98‐100. 15196686 10.1016/j.neulet.2004.04.033

[31] Lee DH , Kim EY , Park S , et al. Reclosure of surgically induced spinal open neural tube defects by the intraamniotic injection of human embryonic stem cells in chick embryos 24 hours after lesion induction. J Neurosurg. 2006;105(2 Suppl):127‐133. 10.3171/ped.2006.105.2.127 16922074

[32] Lee DH , Phi JH , Kim SK , Cho BK , Kim SU , Wang KC . Enhanced reclosure of surgically induced spinal open neural tube defects in chick embryos by injecting human bone marrow stem cells into the amniotic cavity. Neurosurgery. 2010;67(1):129‐135. 20559100 10.1227/01.NEU.0000371048.76494.0F

[33] Li H , Gao F , Ma L , et al. Therapeutic potential of in utero mesenchymal stem cell (MSCs) transplantation in rat foetuses with spina bifida aperta. J Cell Mol Med. 2012;16(7):1606‐1617. 22004004 10.1111/j.1582-4934.2011.01470.x PMC3823228

[34] Li H , Miao J , Zhao G , et al. Different expression patterns of growth factors in rat fetuses with spina bifida aperta after in utero mesenchymal stromal cell transplantation. Cytotherapy. 2014;16(3):319‐330. 24364908 10.1016/j.jcyt.2013.10.005

[35] Ma W , Wei X , Gu H , et al. Sensory neuron differentiation potential of in utero mesenchymal stem cell transplantation in rat fetuses with spina bifida aperta. Birth Defects Res a Clin Mol Teratol. 2015;103(9):772‐779. 26172505 10.1002/bdra.23401

[36] Li X , Yuan Z , Wei X , et al. Application potential of bone marrow mesenchymal stem cell (BMSCs) based tissue‐engineering for spinal cord defect repair in rat fetuses with spina bifida aperta. J Mater Sci Mater Med. 2016;27(4):77. 26894267 10.1007/s10856-016-5684-7 PMC4760996

[37] Wei X , Cao S , Ma W , et al. Intra‐amniotic delivery of CRMP4 siRNA improves mesenchymal stem cell therapy in a rat spina bifida model. Mol Ther Nucleic Acids. 2020;20:502‐517. 32330869 10.1016/j.omtn.2020.03.007 PMC7177192

[38] Wei X , Ma W , Gu H , et al. Transamniotic mesenchymal stem cell therapy for neural tube defects preserves neural function through lesion‐specific engraftment and regeneration. Cell Death Dis. 2020;11:523. 32655141 10.1038/s41419-020-2734-3 PMC7354991

[39] Dionigi B , Ahmed A , Brazzo J, 3rd , Connors JP , Zurakowski D , Fauza DO . Partial or complete coverage of experimental spina bifida by simple intra‐amniotic injection of concentrated amniotic mesenchymal stem cells. J Pediatr Surg. 2015;50(1):69‐73. 25598096 10.1016/j.jpedsurg.2014.10.004

[40] Dionigi B , Brazzo JA, 3rd , Ahmed A , et al. Trans‐amniotic stem cell therapy (TRASCET) minimizes Chiari‐II malformation in experimental spina bifida. J Pediatr Surg. 2015;50(6):1037‐1041. 25929798 10.1016/j.jpedsurg.2015.03.034

[41] Feng C , Christopher DG , Connors JP , Brazzo J, 3rd , Zurakowski D , Fauza DO . A comparison between placental and amniotic mesenchymal stem cells for transamniotic stem cell therapy (TRASCET) in experimental spina bifida. J Pediatr Surg. 2016;51(6):1010‐1013. 27013425 10.1016/j.jpedsurg.2016.02.071

[42] Shieh HF , Ahmed A , Rohrer L , Zurakowski D , Fauza DO . Donor mesenchymal stem cell linetics after transamniotic stem cell therapy (TRASCET) for experimental spina bifida. J Pediatr Surg. 2018;53(6):1134‐1136. 29580785 10.1016/j.jpedsurg.2018.02.067

[43] Lazow SP , Tracy Sarah A , Chalphin AV , Kycia I , Zurakowski D , Fauza DO . Initial Mechanistic Screening of Transamniotic Stem Cell Therapy in the Rodent Model of Spina Bifida: Host Bone Marrow and Paracrine Activity. Fetal Diagnosis and Therapy. 2020;47(12):902–911. 10.1159/000509244. 32877907

[44] Abe Y , Ochiai D , Masuda H , et al. In utero amniotic fluid stem cell therapy protects against myelomeningocele via spinal cord coverage and hepatocyte growth factor secretion. Stem Cells Transl Med. 2019;8(11):1170‐1179. 31407874 10.1002/sctm.19-0002 PMC6811697

[45] Kajiwara K , Tanemoto T , Wada S , et al. Fetal therapy model of myelomeningocele with three‐dimensional skin using amniotic fluid cell‐derived induced pluripotent stem cells. Stem Cell Rep. 2017;8(6):1701‐1713. 10.1016/j.stemcr.2017.05.013 PMC5470234 28591652

[46] Shieh HF , Tracy SA , Hong CR , et al. Transamniotic stem cell therapy (TRASCET) in a rabbit model of spina bifida. J Pediatr Surg. 2019;54(2):293‐296. 30518492 10.1016/j.jpedsurg.2018.10.086

[47] Fauza DO , Jennings RW , Teng YD , Snyder EY . Neural stem cell delivery to the spinal cord in an ovine model of fetal surgery for spina bifida. Surgery. 2008;144(3):367‐373. 18707035 10.1016/j.surg.2008.05.009

[48] Turner CG , Pennington EC , Gray FL , Ahmed A , Teng YD , Fauza DO . Intra‐amniotic delivery of amniotic‐derived neural stem cells in a syngeneic model of spina bifida. Fetal Diagn Ther. 2013;34(1):38‐43. 23635813 10.1159/000350267

[49] Saadai P , Wang A , Nout YS , et al. Human induced pluripotent stem cell‐derived neural crest stem cells integrate into the injured spinal cord in the fetal lamb model of myelomeningocele. J Pediatr Surg. 2013;48(1):158‐163. 23331809 10.1016/j.jpedsurg.2012.10.034

[50] Wang A , Brown EG , Lankford L , et al. Placental mesenchymal stromal cells rescue ambulation in ovine myelomeningocele. Stem Cells Transl Med. 2015;4(6):659‐669. 25911465 10.5966/sctm.2014-0296 PMC4449103

[51] Brown EG , Keller BA , Lankford L , et al. Age does matter: a pilot comparison of placenta‐derived stromal cells for in utero repair of myelomeningocele using a lamb model. Fetal Diagn Ther. 2016;39(3):179‐185. 26159889 10.1159/000433427

[52] Kabagambe S , Keller B , Becker J , et al. Placental mesenchymal stromal cells seeded on clinical grade extracellular matrix improve ambulation in ovine myelomeningocele. J Pediatr Surg. 2017;53(1):178–182. 10.1016/j.jpedsurg.2017.10.032 29122293

[53] Chen YJ , Chung K , Pivetti C , et al. Fetal surgical repair with placenta‐derived mesenchymal stromal cell engineered patch in a rodent model of myelomeningocele. J Pediatr Surg. 2017;53(1):183–188. 10.1016/j.jpedsurg.2017.10.040 29096888

[54] Vanover M , Pivetti C , Lankford L , et al. High density placental mesenchymal stromal cells provide neuronal preservation and improve motor function following in utero treatment of ovine myelomeningocele. J Pediatr Surg. 2019;54(1):75‐79. 30529115 10.1016/j.jpedsurg.2018.10.032 PMC6339576

[55] Galganski LA , Kumar P , Vanover MA , et al. In utero treatment of myelomeningocele with placental mesenchymal stromal cells ‐ selection of an optimal cell line in preparation for clinical trials. J Pediatr Surg. 2019;55(9):1941–1946. 31672407 10.1016/j.jpedsurg.2019.09.029 PMC7170747

[56] Lee DE , Ayoub N , Agrawal DK . Mesenchymal stem cells and cutaneous wound healing: novel methods to increase cell delivery and therapeutic efficacy. Stem Cell Res Ther. 2016;7:37. 26960535 10.1186/s13287-016-0303-6 PMC4784457

[57] Hu MS , Borrelli MR , Lorenz HP , Longaker MT , Wan DC . Mesenchymal Stromal Cells and Cutaneous Wound Healing: A Comprehensive Review of the Background, Role, and Therapeutic Potential. Stem Cells International. 2018;2018:1–13. 10.1155/2018/6901983. PMC5985130 29887893

[58] Harding J , Roberts RM , Mirochnitchenko O . Large animal models for stem cell therapy. Stem Cell Res Ther. 2013;4(2):23. 23672797 10.1186/scrt171 PMC3706788

[59] Liang X , Ding Y , Zhang Y , Tse HF , Lian Q . Paracrine mechanisms of mesenchymal stem cell‐based therapy: current status and perspectives. Cell Transpl 2014;23(9):1045‐1059. 10.3727/096368913X667709 23676629

[60] Cofano F , Boido M , Monticelli M , et al. Mesenchymal stem cells for spinal cord injury: current options, limitations, and future of cell therapy. Int J Mol Sci. 2019;20(11). 10.3390/ijms20112698 PMC6600381 31159345

[61] Dugas A , Larghero J , Zerah M , Jouannic JM , Guilbaud L . Cell therapy for prenatal repair of myelomeningocele: a systematic review. Curr Res Transl Med. 2020;68(4):183‐189. 32624428 10.1016/j.retram.2020.04.004

[62] Hamburger V , Hamilton HL . A series of normal stages in the development of the chick embryo 1951. Dev Dyn. 1992;195(4):231‐272. 1304821 10.1002/aja.1001950404

[63] Brown EG , Keller BA , Pivetti CD , et al. Development of a locomotor rating scale for testing motor function in sheep. J Pediatr Surg. 2015;50(4):617‐621. 25840074 10.1016/j.jpedsurg.2015.01.002

