# Supplementary material for: Preclinical stem cell therapy in fetuses with myelomeningocele: A systematic review and meta‐analysis
Source: Prenat Diagn. 2021 Jan 11;41(3):283–300. doi: 10.1002/pd.5887 (PMC7611444; doi:10.1002/pd.5887)
Supplement: Supplementary file 1 — Supplementary Material [file PD-41-283-s001.docx]

**Supplementary information 1**: search strategy included both Medical Subject Headings (MeSH) term and free text words

“spinal dysraphism” [MeSH] OR “Arnold-Chiari malformation” [MeSH] OR “meningomyelocele” [MeSH] OR spinal dysraphism OR myelomeningocele OR Arnold-Chiari malformation OR meningomyelocele

AND

“stem cells” [MeSH] OR “cell- and tissue-based therapy” [MeSH] OR “stem cell transplantation” OR “cell transplantation” [MeSH] OR “cell injection” [MeSH] OR cell* adj2 therapy OR stem cell* OR cell transplant* OR cell injection

**Supplementary information 2.** Extracted information from included studies and results

| *Extracted information* |
| --- |
| Study and methodology characterisation   - First author name - Title of the paper - Year of publication - Number of animals - Sample randomization |
| Stem cell characterisation   - Specie of the donor - Type of stem cells - Organ that stem cells were extracted from - Control group |
| Animal model   - Specie of recipient - How the MMC lesion was created - Timing of lesion induction - Stem cell application: timing, dosage, timing of transplantation, delivery vehicle - Timing at euthanasia |
| Study results   - Survival rate of the fetuses - Gross examination: rates of defect coverage, neurological function - Histology examination: cross-sectional area of the spinal cord, density of large motor neurons |
